# Endurance tests capture complementary dimensions of physical performance

**DOI:** 10.3389/fphys.2026.1973746

**Published:** 2026-10-01

**Authors:** Petr Kellner, Michal Mašek, Jiří Neubauer, Michal Polách, Michal Punčochář, Jiří Zháněl

**Affiliations:** 1 Centre of Physical Education and Sport, University of Defence, Brno, Czechia; 2 Department of Sport Performance and Exercise Testing, Faculty of Sports Studies, Masaryk University, Brno, Czechia; 3 Department of Military Physical Education, Faculty of Physical Education and Sport, Charles University, Prague, Czechia; 4 Department of Quantitative Methods, University of Defence, Brno, Czechia

**Keywords:** endurance testing, multidimensional assessment, performance profiling, physical performance, principal component analysis

## Abstract

**Introduction:**

Commonly used endurance tests are often treated as interchangeable, although the extent to which they capture shared and distinct dimensions of physical performance remains unclear. This study examined the informational value of commonly used endurance tests and identified a reduced battery that preserved multidimensional performance information.

**Methods:**

A total of 157 men (20.9 ± 1.2 years) and 29 women (20.7 ± 1.0 years) completed six endurance tests alongside assessments of strength, power, agility, and body composition. Correlation analysis, principal component analysis, and multiple regression were used to evaluate shared and unique information across tests.

**Results:**

Running tests were strongly interrelated, as were rowing tests. A general endurance component explained 59.4% and 70.5% of variance in men and women, respectively, while additional components distinguished running, rowing, and physical work capacity. Women exhibited stronger relationships among endurance and other physical performance characteristics. A four-test battery reconstructed 67.6% and 73.5% of the information contained in the full battery in men and women, respectively.

**Conclusion:**

Common endurance tests provide complementary rather than interchangeable information, and a reduced battery can efficiently characterize multidimensional physical performance while substantially reducing testing demands.

## 1 Introduction

Physical fitness testing is widely used for performance evaluation, training monitoring, and verification of occupational standards. Due to their practicality and accessibility, field-based tests are commonly applied in sports clubs, educational settings, and institutions such as the armed forces (Castro-Piñero et al., 2021; Çemç and Şahin, 2024). Within physical fitness assessment, strength and endurance represent major areas of interest, with endurance historically receiving substantial attention in both research and applied practice (Herrador-Colmenero et al., 2014). Numerous field-based endurance tests have demonstrated acceptable validity and reliability for estimating aerobic performance (Castro-Piñero et al., 2021; Cuenca-García et al., 2022), however, the broader informational value provided by individual testing protocols remains insufficiently understood. Despite the widespread use of endurance testing, substantial variation exists in the selection of testing protocols across sports organizations, educational institutions, and military systems, with different tests often employed for the same assessment purpose. This diversity suggests an implicit assumption that alternative protocols may provide comparable information; however, the extent to which commonly used endurance tests reflect similar or distinct aspects of physical performance remains unclear. Consequently, selecting the most appropriate test for a given purpose remains challenging.

Commonly used endurance performance tests differ in their protocol structure, duration, and mode of locomotion, which may result in distinct physiological and performance demands. Continuous running tests such as the Cooper 12-minute run (Cooper, 1985; Bandyopadhyay, 2015), 1000 m run (Díaz et al., 2000), and progressive incremental tests such as the Beep test (Stickland et al., 2003; Mayorga-Vega et al., 2015; Tomkinson et al., 2016) are commonly used as indicators of aerobic performance, whereas rowing-based tests (Funch et al., 2021; Kellner et al., 2024), also used for this purpose, additionally impose substantial upper- and lower-body muscular endurance and strength demands (Cerasola et al., 2020; Kellner et al., 2024). Similarly, cycle-based submaximal protocols such as PWC170 (Boreham et al., 1990) differ substantially from maximal running or rowing tests in their physiological and performance demands (Boreham et al., 1990; Drozd et al., 2025). As a consequence, these tests may not represent interchangeable measures of a single underlying construct and may instead provide partially distinct information about an individual’s physiological and performance profile.

The purpose of this study was to examine the interrelationships and informational value of commonly used endurance performance tests in order to better understand their role in performance profiling. Specifically, the study investigated (i) the strength of associations among six commonly used endurance performance tests, including running-, rowing-, and cycle-based protocols, (ii) the extent to which each test reflects different aspects of an individual’s physiological and performance profile, and (iii) the minimal combination of tests capable of explaining the largest proportion of variance in overall physiological and performance characteristics. By addressing these questions, the study sought to clarify how closely these tests align with the construct of endurance performance, the distinct physiological and performance aspects captured by each test, and whether a minimal test combination can more comprehensively represent overall performance characteristics.

## 2 Materials and methods

### 2.1 Participants

The study sample comprised 157 male (age 20.90 ± 1.17 years, body height 181.28 ± 6.93 cm, body weight 80.80 ± 9.63 kg) and 29 female soldiers (age 20.66 ± 0.97 years, body height 166.79 ± 7.08 cm, body weight 63.87 ± 7.79 kg) who were first-year students at a military university. All participants were healthy, physically active, and accustomed to regular physical fitness training and testing. The sample therefore represented a physically fit young-adult population, with endurance performance approaching the excellent level according to established reference norms. Although rowing was not a regular component of their physical training, all participants had previous experience with rowing ergometer testing and had completed at least one rowing test before participation in the study.

### 2.2 Sample size estimation and justification

All eligible first-year students were invited to participate; therefore, the sample size was determined by the predefined accessible population rather than by an *a priori* power-based recruitment target. The final sample comprised 186 participants (157 men and 29 women).

### 2.3 Study design

Data collection was conducted over a three-week period and comprised six testing days. One day was allocated to anthropometric assessment, spirometry, handgrip strength assessment, and the Physical Working Capacity test (PWC170); one day to Standing Broad Jump, Medicine Ball Throw, and Cooper 12-minute run test (12CT); one day to the Illinois Agility Test and Multistage Fitness Test (Beep test); one day to pull-ups/pull-up hold and the 1000 m run; and two days to the 12-minute rowing test (12RT) and 6-minute rowing test (6RT). Participants were randomly assigned to groups that completed the testing sessions in different orders.

### 2.4 Procedures

For this study, anthropometric data were collected using the InBody 970 (InBody Co., Ltd., InBody Bldg, 625 Eonju-ro, Gangnam-gu, Seoul, 06106, Republic of Korea). Pulmonary function was assessed using a SpiroSonic spirometer (Uscom Kft., Budapest, Hungary), and handgrip strength was measured using a SH5001 hand dynanometer (Saehan Corporation, Changwon, Republic of Korea). The Physical Working Capacity test (PWC170) was performed using a Tacx Neo Bike ergometer (Taxc B.V., Wassenaar, Netherlands). The 12-minute rowing test (12RT) and 6-minute rowing test (6RT) were conducted on a Concept2 Model E rowing ergometer (Concept2 Inc., Morrisville, VT, USA). The Cooper 12-minute run test (12CT) and the 1000 m run were performed on a 300 m athletics track. The standing broad jump, medicine ball throw, Illinois agility test, multistage fitness test (Beep test), pull-ups, and pull-up hold assessments were conducted in an indoor sports hall. All tests were administered by physical education professionals from the university. Participants were instructed to avoid strenuous physical activity before testing sessions and exert maximal effort during all performance tests.

### 2.5 Test protocols

Body composition variables were obtained from bioelectrical impedance analysis. Pulmonary function was evaluated through forced vital capacity (FVC) and forced expiratory volume in one second (FEV1). Handgrip strength (Innes, 1999), Standing Broad Jump (Council of Europe, 1988), Illinois Agility Test (Getchell, 1979), Cooper 12-minute run test (12CT) (Cooper, 1985), Multistage Fitness Test (Beep test) (Léger et al., 1988), Physical Working Capacity test (PWC170) (Boreham et al., 1990), 6-minute rowing test (6RT) (Funch et al., 2021), and 12-minute rowing test (12RT) (Kellner et al., 2024) were performed according to established procedures. Handgrip strength was expressed as the arithmetic mean of the left- and right-hand measurements. For the 12RT, the rowing ergometer damper was set to level 1. Running performance was additionally assessed using a 1000 m run, with completion time used for analysis. Upper-body explosive power was assessed using a standing overhead medicine ball throw. Participants performed the throw using a 4 kg (men) or 3 kg (women) medicine ball. Jumping was permitted provided that the throwing line was not crossed. The longest valid attempt was recorded. Upper-body strength endurance was assessed using pull-ups (men) and the pull-up hold test (women). Pull-ups were performed using an overhand grip, with the number of correctly completed repetitions recorded. The pull-up hold test was performed with the chin maintained above the bar, and the recorded outcome was the duration of the hold in seconds.

### 2.6 Data analysis

Statistical analyses were performed in R (R Core Team, 2026) using the stats and FactoMineR packages (Le et al., 2008). Descriptive statistics, including means, standard deviations, minima, and maxima, were calculated for all variables. Relationships among endurance test outcomes were examined using Pearson’s product–moment correlation coefficient and Spearman’s rank correlation coefficient to assess linear and monotonic associations, respectively, see for example Montgomery & Runger (Montgomery and Runger, 2011). The magnitude of correlation coefficients was interpreted according to Hopkins (2016). To reduce data dimensionality and identify latent performance dimensions, Principal Component Analysis (PCA) was conducted on standardized variables (z-scores), ensuring comparability among measures expressed in different units and scales. Principal component analyses were conducted separately by sex using complete cases and a Spearman correlation matrix. The number of retained components was determined based on the proportion of explained variance and component interpretability (Johnson and Wichern, 2014). An initial PCA was performed on the six endurance tests. Redundancy among endurance tests was evaluated jointly based on inter-test correlations and similarity of their representation in the principal component structure, without applying a predefined numerical threshold. The running-based tests showed substantial overlap, as did the two rowing-based tests; therefore, one representative test from each modality was retained (12CT and 12RT, respectively). Within each modality, the representative test was selected based on the combined pattern of inter-test correlations and PCA loadings. PWC170 was retained because its distinct component structure indicated that it provided information not adequately represented by the running- and rowing-based tests. The three retained endurance tests (12CT, 12RT, and PWC170) were then combined with the five non-endurance performance tests to form an eight-variable reduced battery, on which a second PCA was performed. Following PCA of the reduced test battery, candidate mini-batteries were selected to represent the identified principal performance dimensions while minimizing redundancy among tests. Principal component scores derived from the reduced battery served as dependent variables in multiple regression analyses, and the candidate mini-batteries were entered as predictors. The resulting models were used to quantify the predictive contribution and informational value of each candidate battery with respect to the principal performance dimensions, thereby identifying the smallest battery capable of reconstructing the multidimensional performance structure. To quantify overall reconstruction, a reconstruction index (Rec, %) was calculated as 100 × the variance-weighted mean of the component-specific R² values, using the proportion of total variance explained by each principal component as weights. Thus, Rec (%) represents the percentage of the overall principal component structure reconstructed by a candidate mini-battery, with components explaining more variance contributing proportionally more to the index. The overall analytical procedure is summarized in **Figure 1**. To facilitate interpretation of correlation and principal component analyses, 1000 m and Illinois Agility Test performances were multiplied by −1 prior to analysis so that higher values consistently represented superior performance across all tests.

**Figure 1:**
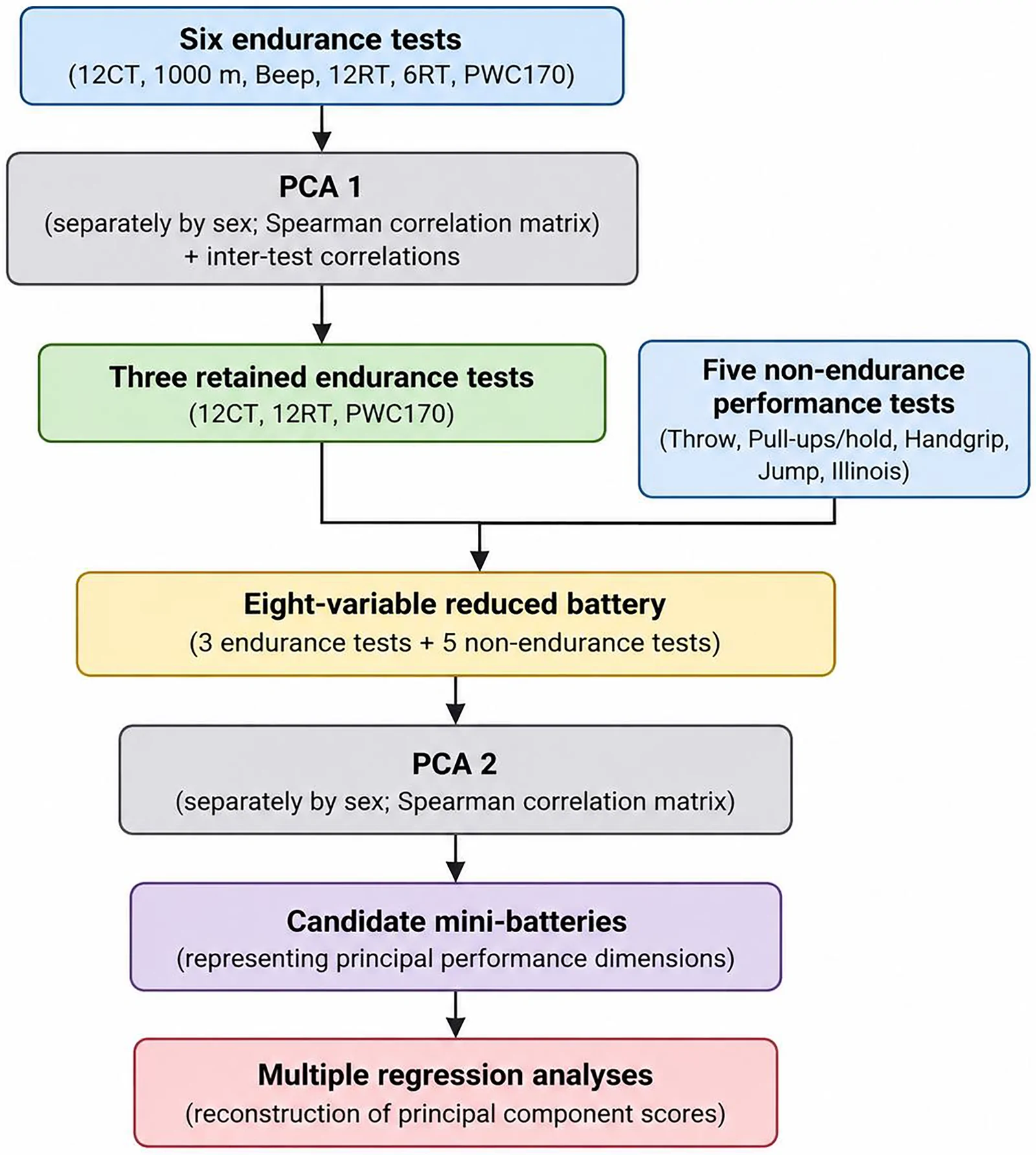
Overview of the statistical analysis and test-battery reduction procedure.

### 2.7 Ethics

The study was conducted in accordance with the ethical standards of the Declaration of Helsinki. The study was approved by the Ethical Board of the university. All participants were informed about the objectives, procedures, and potential risks of the study and provided informed consent before participation.

## 3 Results

Descriptive statistics for the primary study variables are presented in **Table 1**.

**Table 1:** Descriptive statistic characteristics of measured variables.

Discipline	Mean	SD	Med	Min	Max	CV (%)
M 12CT (m)	2803.8	197.1	2780	2350	3500	7.0
W 12CT (m)	2490.0	218.3	2400	2000	2900	8.6
M 1000 m (s)	210.2	14.6	210	177	261	6.9
W 1000 m (s)	239.9	20.0	241	200	264	8.2
M Beep test (s)	524.3	94.2	520	253	750	17.9
W Beep test (s)	406.0	85.9	383	230	580	20.8
M 12RT (m)	2818.0	206.4	2813	2292	3391	7.3
W 12RT (m)	2506.1	268.8	2430	2084	3056	10.5
M 6RT (m)	1520.3	104.4	1520	1248	1762	6.9
W 6RT (m)	1362.1	110.8	1330	1208	1650	8.0
M PWC170 (W/kg)	2.9	0.7	2.8	1.7	5.4	22.4
W PWC170 (W/kg)	2.4	0.7	2.3	1.2	4.7	26.9
M Throw (m)	8.8	1.4	8.8	5.6	13.1	15.9
W Throw (m)	7.6	1.1	7.4	5.7	10.2	14.0
M Pull-up (rep)	12	4.7	12	6	24	38.9
W Pull-up hold (s)	27.9	14.2	28	5	61	49.8
M Handgrip (kg)	50.9	10.9	49.7	20.2	77.9	19.9
W Handgrip (kg)	33.8	9.4	31.3	24.9	63.8	27.4
M Jump (m)	2.3	0.2	2.3	1.7	2.8	10.2
W Jump (m)	2.0	0.3	2.0	1.5	2.4	12.4
M Illinois test (s)	17.6	1.1	17.6	15.2	21.0	6.2
W Illinois test (s)	18.8	1.0	18.6	17.1	21.1	5.5

M, men; W, women; Mean, arithmetic mean; SD, sample standard deviation; med, median; min, minimum; max, maximum; CV, coefficient of variation; 12CT, Cooper12-minute run test; 12RT , 12-minute rowing test; 6RT , 6-minute rowing test; PWC170, Physical Working Capacity test.

Correlations among the primary study variables are presented in **Table 2**.

**Table 2:** Spearman correlation analysis of measured variables.

Discipline	12CT	1000m	Beep	12RT	6RT	PWC170	Throw	Pulls	Handgrip	Jump	Illinois
12CT	—	0.93	0.87	0.77	0.76	0.67	0.54	0.29	0.61	0.70	0.32
1000m	0.73	—	0.85	0.81	0.79	0.67	0.55	0.31	0.63	0.79	0.30
Beep	0.74	0.63	—	0.71	0.75	0.67	0.55	0.19	0.54	0.66	0.43
12RT	0.46	0.47	0.41	—	0.96	0.50	0.70	0.11	0.61	0.66	0.33
6RT	0.44	0.42	0.33	0.82	—	0.54	0.73	0.11	0.67	0.67	0.33
PWC170	0.53	0.44	0.49	0.30	0.28	—	0.25	0.33	0.43	0.46	0.15
Throw	0.04	0.20	0.12	0.34	0.37	0.14	—	-0.15	0.66	0.64	0.25
Pulls	0.19	0.15	0.21	-0.03	-0.06	0.25	-0.07	—	0.07	0.13	-0.33
Handgrip	0.10	0.16	0.08	0.42	0.50	0.09	0.46	-0.07	—	0.72	0.12
Jump	0.19	0.36	0.31	0.32	0.27	0.20	0.48	0.10	0.39	—	0.39
Illinois	0.34	0.34	0.44	0.25	0.21	0.21	0.33	0.04	0.24	0.52	—

Upper triangle , women; lower triangle, men.

Correlations among the measured variables are presented in **Table 2**. In men, the strongest correlations were observed among the running-based endurance tests (r = 0.63–0.74) and between the two rowing tests (r = 0.82). In women, running-based endurance tests were more strongly correlated (r = 0.85–0.93), while the two rowing tests showed an almost perfect correlation (r = 0.96). Correlations between endurance and strength-related measures were generally higher in women than in men.

Principal component analysis of the endurance tests identified three principal components explaining 87.6% and 93.6% of the total variance in men and women, respectively. The first component explained 59.4% (men) and 70.5% (women) of the variance. In men, the second and third components explained 17.7% and 10.5% of the variance, respectively, whereas in women they explained 14.6% and 8.5%. Correlations between endurance tests and principal components are presented in **Table 3**.

**Table 3:** Correlations between endurance tests and principal components.

Discipline	PC1	PC2	PC3	PC4	PC5	PC6
M 12CT (m)	0.88	-0.24	-0.15	-0.11	-0.32	-0.17
W 12CT (m)	0.91	0.04	-0.27	-0.17	0.24	-0.06
M 1000 m (s)	0.84	-0.15	-0.26	-0.38	0.23	0.073
W 1000 m (s)	0.93	-0.05	-0.12	-0.23	-0.25	0.03
M Beep test (s)	0.77	-0.35	-0.26	0.43	0.06	0.11
W Beep test (s)	0.87	0.04	-0.39	0.30	-0.04	0.05
M 12RT (m)	0.76	0.56	0.06	0.14	0.17	-0.24
W 12RT (m)	0.88	-0.27	0.36	0.01	0.10	0.16
M 6RT (m)	0.73	0.61	0.14	-0.02	-0.15	0.24
W 6RT (m)	0.90	-0.20	0.32	0.11	-0.04	-0.17
M PWC170 (W/kg)	0.61	-0.42	0.67	-0.01	0.05	0.00
W PWC170 (W/kg)	0.44	0.87	0.21	0.01	-0.00	0.01

PC, Principal Component.

Based on the correlation and principal component analyses, the Cooper 12-minute run, 12-minute rowing test, and PWC170 were retained for subsequent analyses, whereas the Beep test, 1000 m run, and 6-minute rowing test were excluded because they provided largely redundant information.

Principal component analysis of the reduced test battery showed that the first four components explained 77.6% of the total variance in men (36.6%, 18.7%, 13.0%, and 9.3%, respectively) and 83.9% in women (42.9%, 19.8%, 12.0%, and 9.3%, respectively). Correlations between the reduced battery tests and principal components are presented in **Table 4**.

**Table 4:** Correlations between reduced battery tests and principal components.

Discipline	PC1	PC2	PC3	PC4	PC5	PC6	PC7	PC8
M 12CT (m)	0.55	0.61	-0.35	-0.2	0.12	0.03	0.01	-0.37
W 12CT (m)	0.79	0.14	0.35	-0.27	-0.03	-0.26	-0.26	0.16
M 12RT (m)	0.68	0.08	-0.53	0.13	0.18	-0.36	-0.02	0.27
W 12RT (m)	0.82	0.01	0.08	-0.44	0.23	0.03	0.11	-0.27
M PWC170 (W/kg)	0.53	0.62	0.04	0.05	-0.48	0.25	-0.06	0.2
W PWC170 (W/kg)	0.35	0.78	0.08	-0.05	-0.46	0.21	0.03	-0.04
M Throw (m)	0.69	-0.4	0.12	0.24	-0.36	-0.22	0.3	-0.18
W Throw (m)	0.73	-0.41	-0.34	-0.12	0.05	0.36	-0.13	0.15
M Pull-up (rep)	0.14	0.59	0.63	0.33	0.32	-0.12	0.12	0.03
W Pull-up hold (s)	0.23	0.81	-0.07	0.24	0.46	0.06	0.02	0.09
M Handgrip (kg)	0.64	-0.37	-0.13	0.39	0.26	0.46	-0.01	-0.01
W Handgrip (kg)	0.79	-0.04	-0.43	0.06	-0.13	-0.23	0.32	0.11
M Jump (m)	0.74	-0.24	0.39	-0.1	-0.01	-0.14	-0.46	-0.08
W Jump (m)	0.75	-0.11	-0.14	0.55	-0.07	-0.08	-0.21	-0.19
M Illinois test (s)	0.66	-0.14	0.24	-0.6	0.16	0.1	0.24	0.15
W Illinois test (s)	0.49	-0.33	0.7	0.31	0.02	0.13	0.2	0.07

PC, Principal Component.

Several candidate mini-batteries were evaluated using multiple regression analysis. Principal component scores derived from the reduced battery served as dependent variables, and the candidate test batteries as predictors.

Among the three-test solutions, the Cooper 12-minute run, standing long jump, and pull-ups provided the highest reconstruction in men (56.0%), whereas the Cooper 12-minute run, medicine ball throw, and pull-up hold performed best in women (62.8%). The highest reconstruction was achieved by the four-test battery consisting of the Cooper 12-minute run, medicine ball throw, pull-ups/pull-up hold, and Illinois agility test, reconstructing 67.6% and 73.5% of full battery information in men and women, respectively (**Table 5**).

**Table 5:** Reconstruction of the principal component structure by the optimal minimalist test batteries.

Test batteries	PC1	PC2	PC3	PC4	PC5	PC6	PC7	PC8	Rec (%)
Men: 12CT + jump + pull-ups	0.74	0.77	0.76	0.19	0.11	0.03	0.25	0.15	56.0
Men: 12CT + throw + pull-ups + Illinois test	0.84	0.82	0.75	0.75	0.32	0.11	0.13	0.28	67.6
Women: 12CT + throw + pull-up hold	0.87	0.78	0.45	0.17	0.26	0.37	0.07	0.04	62.8
Women: 12CT + throw + pull-up hold + Illinois test	0.89	0.84	0.85	0.46	0.28	0.44	0.21	0.04	73.5

PC, principal component; values in PC1–PC8 columns represent R² for individual principal components. Rec (%), reconstruction index calculated as the variance-weighted mean of the component-specific R² values, expressed as a percentage; weights correspond to the proportion of total variance explained by each principal component.

## 4 Discussion

The present study examined the informational value of six commonly used endurance performance tests and explored the feasibility of constructing a minimalist test battery capable of representing broader performance characteristics. The main findings and their implications are discussed below.

The correlation and principal component analyses demonstrated that the endurance tests captured both shared and unique aspects of endurance performance. Strong correlations were observed among the running-based endurance tests, ranging from r = 0.63–0.74 in men and r = 0.85–0.93 in women, which is consistent with previous evidence demonstrating that various run/walk field tests provide valid estimates of cardiorespiratory fitness (Mayorga-Vega et al., 2016). Similarly, the two rowing tests exhibited very strong associations in both sexes (men: r = 0.82; women: r = 0.96). Correlations between running- and rowing-based endurance tests were generally moderate in men (r = 0.33–0.47) and large to very large in women (r = 0.71–0.81), supporting previous studies (Kellner et al., 2024). The relationship of PWC170 with the remaining endurance tests was moderate in men (r = 0.28–0.53) and large in women (r = 0.50–0.67). Principal component analysis supported these findings, identifying a dominant general endurance factor that explained 59.4% and 70.5% of the total variance in men and women, respectively. At the same time, additional components differentiated rowing-based tests from running-based tests and identified PWC170 as a distinct physiological dimension. Together, these findings indicate that endurance tests share a substantial common component but are not fully interchangeable.

Despite the substantial overlap among endurance tests, their ability to represent the overall endurance construct differed. In men, the Cooper 12-minute run demonstrated the strongest overall relationships with the remaining endurance tests, followed by the 1000 m run, suggesting that these running-based protocols provided the most comprehensive representation of endurance performance. In women, the 1000 m run exhibited the strongest overall associations, closely followed by the Cooper test. Conversely, PWC170 demonstrated the weakest relationships with the remaining endurance tests in both sexes and emerged as a distinct principal component. This finding may be related to the submaximal nature of the PWC170 protocol, which estimates performance indirectly from the heart rate response to exercise rather than from maximal performance itself. Consequently, PWC170 appears to capture a partly distinct aspect of endurance performance compared with the maximal field tests included in the present study.

A notable finding was the substantially stronger alignment among endurance tests observed in women. The principal component analysis revealed a comparable pattern, with the general endurance component accounting for 70.5% variance in women and only for 59.4% in men. Furthermore, the first three principal components explained 93.6% of the total variance in women compared with 87.6% in men. These findings suggest a more unified structure of endurance performance in women within the present sample. However, given the substantially smaller female sample, this sex-related difference should be interpreted cautiously. The observed pattern suggests that individual endurance testing protocols may be more interchangeable in women, whereas combining multiple endurance modalities may provide greater additional information in men.

The broader correlation analysis demonstrated that endurance tests differed considerably in the additional information they provided beyond endurance performance itself. In men, running-based endurance tests showed only weak relationships with strength and power measures, with most correlations below r = 0.30, whereas rowing-based endurance tests demonstrated moderate associations with the strength spectrum. These findings suggest that rowing performance reflects not only aerobic endurance but also a meaningful contribution of upper-body strength and power characteristics (Cerasola et al., 2020). In women, both running- and rowing-based endurance tests demonstrated substantially stronger relationships with strength and power measures (r = 0.54 – 0.73), further supporting the notion that physical performance characteristics are consistent with the pattern of stronger interrelationships observed in the female subgroup.

Body composition demonstrated a similar pattern. In men, only a limited number of associations exceeded |r| = 0.30, primarily involving pull-up performance and running-based endurance tests. In contrast, women demonstrated substantially stronger and more widespread associations, with higher skeletal muscle percentage generally associated with superior performance and higher adiposity associated with poorer performance. The strongest relationships were observed for pull-up hold performance and running-based endurance tests, where correlations frequently exceeded |r| = 0.40 and approached |r| = 0.60. These findings are consistent with the pattern of stronger interrelationships observed in the female subgroup and with previous studies identifying body composition, particularly adiposity, as an important determinant of endurance and strength performance (Arrese and Ostáriz, 2006; Harman et al., 2008).

The reduction of the endurance battery was intended to minimize redundancy while preserving the principal endurance dimensions identified in the endurance-specific analyses. Correlation and principal component analyses indicated that the Beep test, 1000 m run, and 6-minute rowing test provided largely redundant information, whereas the Cooper 12-minute run, 12-minute rowing test, and PWC170 best represented the principal endurance dimensions identified in the original battery. The reduced-battery principal component analysis demonstrated that endurance performance remained an important component of overall physical performance but no longer dominated the component structure. This suggests that the reduction procedure successfully minimized redundancy while preserving the principal performance dimensions contained within the original battery. In men, performance was distributed across several relatively independent dimensions, including general performance, endurance modality, upper-body strength, and agility. In contrast, endurance, strength, and power measures clustered more strongly within a common dimension in women, resulting in a larger first principal component (42.9% vs. 36.6%) and greater cumulative variance explained by the first four components (83.9% vs. 77.6%).

The mini-battery analyses further highlighted the central role of endurance performance within overall physical performance assessment. Regardless of the specific test combination evaluated, the most successful mini-batteries consistently included an endurance measure. Although the Cooper 12-minute run ultimately formed part of the optimal mini-batteries, the 12-minute rowing test demonstrated comparable informational value and, in men, provided a slightly stronger representation of the dominant performance dimension. However, batteries incorporating the Cooper 12-minute run achieved marginally higher overall reconstruction of the multidimensional component structure and were therefore retained in the final solutions. This finding suggests that while the 12-minute rowing test may be an excellent indicator of general performance, the Cooper test captures a slightly broader range of performance characteristics across the entire component structure.

Among the three-test solutions, the combination of the Cooper 12-minute run, standing long jump, and pull-ups provided the best representation of the reduced-battery component structure in men, reconstructing 56.0% of the variance structure. In women, the combination of the Cooper 12-minute run, medicine ball throw, and pull-up hold achieved a reconstruction value of 62.8%. The inclusion of the Illinois agility test substantially improved reconstruction in both sexes. Consequently, the four-test batteries reconstructed 67.6% and 73.5% of the reduced-battery variance structure in men and women, respectively. The consistently higher reconstruction values observed in women further support the findings of the correlation and principal component analyses. Because endurance, strength, power, and agility measures exhibited stronger interrelationships in women, a smaller number of tests was able to capture a larger proportion of the overall performance structure. In contrast, the relatively independent performance dimensions observed in men limited the ability of a small number of tests to represent overall physical performance.

Several limitations should be considered when interpreting the findings of this study. First, the participants represented a relatively fit population. According to established Cooper test norms (Cooper, 1985), the average performance of men (2804 ± 197 m) was located near the boundary between above-average and excellent endurance levels, whereas the average performance of women (2490 ± 218 m) corresponded to an above-average endurance level. Similarly, handgrip strength values in both sexes were above normative reference values reported for comparable age groups (Wang et al., 2018). Consequently, the observed relationships among tests may not fully generalize to less physically active populations, and the observed sex differences may have been influenced, at least in part, by differences in the fitness level of the examined groups.

Second, the substantially smaller female sample (n = 29) compared with the male sample (n = 157) limits the precision and stability of estimates in women, particularly in multivariate analyses. Consequently, the observed sex-related differences should be interpreted cautiously and require confirmation in larger female samples.

Third, the proposed minimalist test batteries were derived from the specific set of endurance, strength, power, and agility tests included in the present study. While the identified batteries reconstructed a substantial proportion of the multidimensional performance structure (67.6% in men and 73.5% in women), additional performance domains such as sprinting, balance, coordination, flexibility, or sport-specific skills may contribute further independent dimensions. Therefore, the present findings should be viewed as an initial step toward the development of broader evidence-based performance assessment batteries rather than a definitive solution for comprehensive physical performance profiling.

The present findings have important practical implications. The proposed four-test battery provides an efficient alternative to comprehensive fitness testing when time, personnel, or equipment are limited. Furthermore, practitioners should not consider running-, rowing-, and cycle-based endurance tests interchangeable, as each provides partly distinct information about physical performance.

## 5 Conclusions

The present study demonstrated that commonly used endurance performance tests capture both shared and unique aspects of endurance performance. While running-based tests, rowing-based tests, and PWC170 exhibited substantial overlap, principal component analysis revealed distinct dimensions associated with testing modality and physiological characteristics, indicating that these tests are not fully interchangeable. A consistent pattern observed in the present sample was stronger interrelationships among performance characteristics in women than in men. Women exhibited stronger relationships among endurance, strength, power, agility, and body composition measures, resulting in a more unified performance structure and greater reconstruction of the measured performance variables using a reduced number of tests. However, given the substantially smaller female sample, this finding should be interpreted cautiously and requires confirmation in larger female samples. The reduction of redundant endurance protocols allowed the identification of a practical four-test battery consisting of the Cooper 12-minute run, medicine ball throw, pull-ups (or pull-up hold), and the Illinois agility test. This battery reconstructed 67.6% and 73.5% of the information captured by the full test battery in men and women, respectively, while substantially reducing testing demands. These findings suggest that the physical-performance characteristics assessed in this study are multidimensional and cannot be fully represented by a single endurance test. Nevertheless, carefully selected combinations of tests can provide a practical and informative representation of the information captured by a larger test battery while maintaining reasonable testing efficiency.

## Data Availability

The datasets presented in this article are not readily available because The data access is restricted by University of Defence. Requests to access the datasets should be directed to petr.kellner@unob.cz.
